# Barriers to nurses’ therapeutic communication practices in a district hospital in Ghana

**DOI:** 10.1186/s12912-023-01191-2

**Published:** 2023-02-07

**Authors:** Evans Osei Appiah, Ezekiel Oti-Boadi, Mary Ani-Amponsah, Dorcas Goku Mawusi, Dorothy Baffour Awuah, Awube Menlah, Cindy Ofori-Appiah

**Affiliations:** 1 Department of Midwifery, School of Nursing and Midwifery, Valley View University, P.O. Box DT 595, Oyibi, Ghana; 2 School of Nursing and Midwifery, Valley View University, Oyibi, Ghana; 3 Maternal and Child Health Department, School of Nursing and Midwifery/ College of Health Sciences, University of Ghana, Legon, West-Africa Ghana; 4 Department of Nursing, Nursing School of Nursing and Midwifery, Valley View University, Oyibi, Ghana; 5 Ghana Christian University College, Amrahia, Ghana

**Keywords:** Barriers, Nurses, Practices, Therapeutic communication

## Abstract

**Background:**

Patients accessing health care enter the hospital environment with extreme anxiety, fear and distress which impacts their interactions with nurses and other health care professionals who are expected to help allay these anxieties in order to enhance patients care satisfaction. However, evidence suggests that there is a lack of effective therapeutic nurse-patient interaction in hospitals and the clinical environment globally, especially in sub-Saharan Africa.

**Methods:**

A qualitative research approach with an exploratory design was used to purposively select 30 participants who were engaged in face-face interactions. A semi-structured interview guide was used to conduct five audio-recorded FGDs with the 30 participants (6 in each group—2 males and 4 females) after which the discussions were transcribed verbatim, and content analyzed.

**Findings:**

Two (2) main themes and 10 sub-themes emerged from the analysis of the data. The two themes were: *Therapeutic communication practices and Barriers to therapeutic communication*. Some of the factors identified by patients to impede therapeutic nurse-patient interaction include family interference, negative attitude from patients, patient condition, a discriminatory attitude of nurses, increased workload, and stress.

**Conclusion:**

Communication practices identified in this study include nurses’ manner of communication, use of touch, positive reassurance, and nurses’ demeanor. Several obstacles affect communication practices, hence the need to implement measures to improve nurse-patient interaction.

**Supplementary Information:**

The online version contains supplementary material available at 10.1186/s12912-023-01191-2.

## Introduction

Hospitals are established to render comprehensive care to people who are who are ill physically, mentally, or emotionally. It is believed that patients seeking health care have fears and anxieties which may be rooted in the caregiving process, and it is expected that their interactions with the nurses and other health professionals will assuage these anxieties in order to achieve satisfaction [1]. Healthcare workers can effectively calm patients' who are anxious through communication [2]. For communication to be effectively productive, the focus must be placed on how the information is transmitted, especially in nursing practice [3]. The authors further ascertained that therapeutic communication involves making the needs of patients a priority, creating a favorable environment for patients, and involving patients in the care carried out to them.

It is noted that desired patient satisfaction and positive experiences are the gateway to better treatment outcomes [4]. Having excellent therapeutic communication has been linked to patient satisfaction and pleasant experiences [5]. According to the Institute of Medicine, patient-centered care details the provision of care that respects patients’ views, responds to patient’s preferences, needs, and values, and ensures that patient values guide all clinical decisions through therapeutic communication. Therapeutic communication refers to purposeful communication which helps patients to analyze and solve problems [6]. It is aimed at providing emotional support to patients to improve patient outcomes. Therapeutic communication involves establishing an interpersonal relationship between the nurse and the patient with the aim of providing patients healing [7]. It is an interaction between health care providers and their patients to promote the physical and emotional well-being of patients [8].

However, ineffective therapeutic communication practices is a commonplace in Africa (Ghana, Ethiopia etc.), compared to the advanced countries which may negatively impact nursing care delivery in these countries [9, 10]. Furthermore, a study finding confirmed the apparent lack of cordiality in nurse-patient interactions [11]. A study in Ghana further identified that the nurse-patient therapeutic relationship depends not only on caregiver attitudes but also on other factors such as hospital wait times and patient comfort [12].

There have been several reports of nurses making derogatory remarks in their interactions with patients in public hospitals. Most research on the nurse-patient relationship focuses on the nurses’ and other healthcare providers’ side of the story, ignoring the patient's opinion almost completely [13–16]. Therapeutic communication is inherent in nursing practice and must be practiced to ensure that quality care is consistently delivered to patients in order to achieve good outcomes [17, 18]. It is therefore essential to examine the therapeutic communication practices among nurses so that, pragmatic recommendations could be adopted to help improve this area of practice.

Despite the usefulness and recognition of therapeutic communication in health care, hospitals around the world, especially in sub-Saharan Africa, lack effective therapeutic interactions between nurses and patients [19, 20]. It is estimated that 27% of medical errors are due to communication inaccuracy [13]. Better communication can reduce medical errors and patient injury. Poor communication can lead to a variety of negative consequences, including poor treatment compliance, safety concerns, patient dissatisfaction, and inefficient use of resources [21].

Effective communication skills are of value in healthcare as they have been shown to reduce medical errors and promote patient outcomes [22]. However, some authors have identified some obstacles hindering the implementation of therapeutic communication in Ghana including patients’ anxiety, pain, language barrier, poor nurse patients relationship, and educational status which were all conducted in the Ashanti Region of Ghana [23–26]. It was also noted that most of these studies adopted a quantitative approach in assessing this phenomenon; the surveys established that challenges with therapeutic communication still exist. Hence the current study aimed to explore practices and barriers to therapeutic communication among nurses at a district hospital in Accra, Ghana.

### Research questions

The research questions formulated were.

A What were the therapeutic communication practices among nurses?
B What are the obstacles to therapeutic nurse-patient communication?

## Methods

### Design and setting

A qualitative approach was employed for this study. The study design was exploratory descriptive as it helps explain, describe and obtain detailed information about the phenomenon [27]. Thus, this design enabled the participants to share their views on what they believe to be barriers to therapeutic communication based on their own experiences and also allowed them to share how they view and practice therapeutic communication. The setting selected was a district hospital located in the Eastern part of the Greater Accra Region of Ghana, West Africa. This setting was selected because it renders services to various kinds of patients who speak different languages making communication with them difficult and this current study is the first to be conducted in this setting.

### Study participants

The target population for this study was professional nurses at a district hospital in Ghana. Hence, professional nurses with at least two years’ work experience were included in this study. Exempted from this study are other healthcare professionals and nursing students having their clinical training in the facility used.

A sample is defined as a group of people, objects, or items that are taken from a larger population for measurement [28]. This study employed a purposive sampling method; with selection based on the qualities of the population studied and the objectives of the study [29]. This sampling technique was deliberately employed to allow researchers, select staff nurses, from the study setting, who have worked for two years or more. Sample size refers to the number of individuals included in a research study to represent a population [30]. Five focused-group discussions were held with six nurses in each group until we achieved data saturation with a sample size of 30. During the fourth FGD, participants were given similar ideas as mentioned by participants in previously conducted interviews. The authors further conducted one more interview and no new information was recounted by the participants, hence data collection was ended after the fifth FGD. In all, 40 participants were contacted, and 10 of them refused to partake in the study due to busy schedules and other personal reasons.

### Data collection instrument and procedure

The data collection tool was developed by researchers based on previous published studies of the phenomenon and the research objectives. The tool had three sub-sections, section A elicited information on the socio-demographic characteristics of the results (Age, sex, marital status, etc.), Section B consisted of questions on barriers to therapeutic communication (For example what are the challenges with therapeutic nurse-patient communication?, what do you find challenging with therapeutic communication during health care delivery?), and Section C had questions of practices of therapeutic communication (Example, What are the strategies you used to ensure therapeutic communication between you and your patients?). The study instrument was first pretested on one FG comprising six nurses at the Valley View University hospital in Ghana. Prior to the data collection, the purpose and procedure for the data collection were explained to the nurses who met the inclusion and they were made to understand that they have the free will to decide whether or not to participate in the study. Following this, in-person FGDs were conducted in English by the researchers at the date, time, and place (at the hospital and home) agreed on by all the respondents in a particular group lasting for 3 months from November 2021 to January 2022. The FGDs consisted of six participants each (2 males and 4 females in each group). Thirty participants took part in the audio-recorded FGD session each lasting from 40 to 60 min.

Participants were given the opportunity to read the consent form after which due questions and concerns were addressed, before signing the consent form prior to the interviews. Covid-19 protocols were strictly adhered to by ensuring appropriate distancing and the use of facemasks. All the authors took part in the data collection. The researchers penned down issues of interest and concern in their diaries during the interview to aid in probing for further clarification. The researchers served as moderators (asking questions and ensuring a productive discussion) during the interviews. Before the interviews, the discussion norms (Not to interrupt others when expressing their views and not to dominate the group) were explained, and participants were allowed to engage freely in the audio-recorded discussions. Participants were also made aware of the need to keep all information during the FGDs confidential. Field notes were taken and a reflective journal was kept to document experiences, participants’ non-verbal communication, and other nuances of relevance to the phenomenon under inquiry.

### Ethical consideration

Ethical clearance was also sought from the Dodowa Health Research Centre Institutional Review Board (DHRCIRB- DHRCIRB120/11/21) of the Ghana Health Service prior to data collection. Also, the study had no potential harm to the participants, and the findings are expected to benefit both patients and nurses. The nurses were told that participation is optional.

### Data analysis

Data analysis is defined as a process of cleaning, transforming, and modeling data to discover useful information for decision-making [31]. Data were content analyzed. Content analysis is a research method used to identify patterns in recorded communication [32]. Content analysis is a method of gathering and analyzing the content of written, verbal or visual communication. The data was content analyzed by the first 4 authors using conventional content analysis following the verbatim transcription through familiarization, condensation, coding, categorization, and generation of themes and sub-themes [33]. The familiarization phase is the first step, where the researchers transcribed the audio files and divided the transcripts among themselves to read severally in order to understand the responses of the participants.

Following this, the researchers grouped words of similar meaning together after which it was condensed (shortened) by using a few sentences of similar meaning to represent these ideas. For instance, one of the sentences condensed was “The way and manner nurses approach their patients”.

Following this, 2–3 words were used to represent (label) the shortened sentences (coding) whilst maintaining the original meaning Wherever in the transcripts nurses’ behaviors whilst communicating with their patients such as anger, smiling, etc. were addressed were replaced/labeled with “Nurse’s mannerisms”.

Related codes were then grouped together by the process known as categorization. All the codes were copied into a one-word document. Repeated codes were deleted. The authors did a reconciliation of some codes that were similar. Communication practices was deemed appropriate as a category for all codes addressing nurses’ behaviors during communication which was further formulated into “therapeutic communication practices” as one of the major themes. Two main themes and 12 subthemes were generated.

### Below is an illustration

Condensation-The way and manner nurses approach their patients.

Coding Nurse’s mannerisms.

Categorization-Communication practices.

Theme- Therapeutic communication practices.

The study was guided by COREQ software which has been uploaded as a supplementary file.

### Trustworthiness

The criteria for ensuring rigor in qualitative research are credibility, dependability, confirmability, and transferability [34]. Credibility is the confidence that can be placed in the truth of research findings. This was achieved by strictly adhering to the research objectives throughout the study and selecting participants using the inclusion criteria. For researchers to be able to replicate this study in different settings, the authors described the methods in detail including the design, sampling techniques, sample size, data collection procedure, and instrument. The results from the study reflected the participant’s perception of the practices of therapeutic communication. Dependability is defined as the stability of findings over time [35]. Dependability and confirmability were achieved by pretesting the data collection tool, recording all participant’s data, and transcribing verbatim audio files.

## Results

### Socio-demographic characteristics of participants

The socio-demographic characteristics examined from the 30 respondents included age, gender, marital status, religion, ethnicity, educational background, and rank. The age of the participants ranged from twenty-one (21) to thirty-five (35) years. The majority (80%) of the participants fell within the age group of 21 years to 30 years., followed by the age range 31 years to 35 years (20%). The analysis of the results showed that out of 30 participants, the percentage of females was 66.6% and the percentage of males was 33.3%. The details are presented in Table 1.

**Table 1:** Socio-Demographic Characteristics of Respondents

Variable	Frequency(*n* = 30)	Percentage (%)
**Age group**		
21–30	24	80
31–35	6	20
**Gender**		
Male	10	33.3
Female	20	66.6
**Religion**		
Christian	29	96.7
Muslim	1	3.3
**Ethnicity**		
Ga-adamgbe	2	6.7
**Ga**	5	16.6
Akan	14	46.7
Ewe	6	20
Fante	1	3.3
Northerner	2	6.6
**Marital status**		
Single	22	73.3
Married	11	26.7
Divorced	0	0
**Educational Status**		
Degree	28	93.3
Masters	2	6.6
**Rank**		
NO	24	60
SNO	4	13.3
PNO	2	6.6

#### Organization of the themes

The two (2) main themes and 10 sub-themes were constructed by the researchers as presented in Table 2.

**Table 2:** Emerged themes and sub-themes

Themes	Subthemes
1. Therapeutic communication practices	1. Mannerisms during communication 2. Use of touch 3. Positive reassurance 4. Maintaining a calm demeanor 5. Involving patient relative
2. Barriers to therapeutic communication	1. Patient condition 2. Negative attitude from patients 3. Increased workload/stress 4. Family interference 5. Discriminatory attitude of nurses

### Therapeutic communication practices

#### Nurses’ manner of communication

The study participants indicated that for therapeutic communication to be effective, the manner in which nurses communicate should be considered. This was depicted in the ensuing verbatim statements;

*‘‘When you start with a smile, they will also respond with a smile even when they are angry, they will respond with a smile but if you go screaming and shouting at them because you wanted them to do something which they are not doing, they will also retaliate. So, it depends on the way and manner you will approach them’’.* (FG 1, N 1)

*‘‘Patients are now more particular about the manner in which nurses communicate rather than the words being spoken. So, whenever they rush a patient to my unit, I quickly meet them and assist them in a bed or chair to make them comfortable and feel at home’’. (*FG5, N6)

Some participants shared their observations about poor nurse-patient interactions used by some student nurses and how they approached them.

*‘‘Couple of times, I have observed some student nurses who were communicating with their patients and chewing gum. When I see that, I call them to my office to advise that they stop since those are not good manners’’. (*FG2, N3)

Some respondents spoke of how they dealt with negative manners of some patients. This is illustrated in the response below;

*‘‘Some patients come in angry because of negative previous experiences and that alone can increase their blood pressure. So as a nurse, I use consoling words that will make them feel we understand what they are going through, and we give them the opportunity to express their concerns to help calm them down’’. (FG5,* N2)

#### Use of touch

The current study participants reported how they used touch to communicate and care for their patients. Below are some of the statements by the respondents on the use of touch in therapeutic communication;

*‘‘We sometimes use touch to know the exact place the client is feeling the pain as well as the severity of the pain since pain is subjective’’* (FG5, N5)

*‘‘Mostly, patients come to the hospital very anxious and in pain. When patients are in pain, it is very difficult to interact with them. So usually, we give them a gentle rub on the back of their hand and tell them that everything is going to be okay.’’.* (FG5, N1)

Some participants verbalized how touch could be used to manage patients with special problems such as depression.

*‘‘Most patients who are depressed do not need too many words. Sometimes, you just have to sit beside them, tap their shoulders and give them a shoulder to cry on. A gentle rub has been proven to help manage pain; once in a while, I do that and it helps a lot. (*FG2, N2)

However, some reported that nurses should be cautious of how they touch patients to avoid misinterpretations.

*One has to be careful how he/she touches the patient, especially if you are a male nurse and the patient is a female in order not to cause sexual arousal or make the patient offended’’.* (FG4. N3)

#### Maintaining a calm demeanor

The participants of the study recounted how nurses should maintain a friendly demeanor when their patients are angry in order to calm them down. Below are some responses to demonstrate this;

*‘‘With angry patients, you don’t also meet them with anger. You calmly explain things to them and tell them that they should calm down since you are ready to give them a listening ear. At that moment the patient might be aggressive, so the nurse needs to be relaxed and calmer’’.* (FG 1, N6)

*‘‘You calm angry patients by first of all being assertive and strict…Err not strict but firm. You have to talk to the patient firmly making sure you exercise some kind of control over the situation and you also have to ensure that other patients are safe and protected from the angry patient.* (FG 3.N3)

Some participants mentioned how they sometimes control their negative emotions in order to provide care to their patients.

*‘‘Sometimes we lose patients we have tried our possible best on and it becomes painful. But we have to control our emotions in order to be able to reassure the relatives’’. (FG5, N1)*

#### Involving patient relatives

The nurses in this study declared how they collaborated with patients relatives to render care to the patients under their care. This is shown in the responses below;

*‘‘Okay, we ensure relatives take part in the care rendered to the patients. So right from admission, we welcome and reassure both the patients and their relatives. We make the aware of the kind of foods allowed and restricted, visiting hours, their medications, etc since they can positively or negatively influence their decision to comply with treatment. (*FG 2, N4)

*‘‘Relatives are made known of hospital routines since they will be doing most of the things for the patient. I usually explain the medical diagnosis or the pathology or the cause of the disease with patient consent’’.* (FG3, N6)

Participants revealed that how patients’ relatives are treated may have an impact on the patient’s well-being and hence suggested the need to treat patient’s relatives with care*.*

*‘‘I have seen most nurses maltreating patient relatives and talking to them harshly, but this can worsen the patient condition, so they should be treated with love for us to work together with them for the benefit of the patient’’.* (FG1, N1)

#### Positive reassurance

The respondents in the study revealed there were times that they had to reassure patients because they were sad. They indicated that positive reassurance is essential in nursing care. Some of these narrations were captured below;

*‘‘As nurses, we have to be positive about every situation, in order to help our clients to also remain hopeful. This helps reduce the anxieties of patients and relatives’’. (*FG4, N2)

*‘‘Being positive should be part of every nurse because if the care providers lose hope, what will the patient relatives do? The only challenge with reassurance is when the condition cannot be cured but even with that, we make them aware of the treatment available to manage these symptoms’’.* (FG3, N3)

Some participants noted that nurses should not give false hope during reassurance.

*‘‘Giving assurance is good but it doesn’t mean you should tell the patient lies or withhold some information from patients. They should know the whole truth and exactly what is happening for them to prepare for any mis happenings’’.* (FG1, N5)

### Barriers to therapeutic communication

#### Patient’s condition

Participants acknowledged that the patient’s condition may interfere with his or her ability to engage in therapeutic communication with the nurse as shown in the responses below.

*‘‘Hmm, sometimes, when patients have been diagnosed with a stroke, it is very difficult to communicate with them. A patient with a stroke won’t be able to communicate with you therapeutically. Sometimes their words do not make meaning or are incoherent making it difficult for the nurse to hear and respond appropriately.’’. (FG5,* N3)

*‘‘Okay, so when the patient is unconscious, it becomes very difficult to communicate with him or her. An unconscious patient is unable to speak for himself or herself. The relatives are the ones who help. But if the relatives are not around, it becomes very difficult to communicate with the patient’’. (FG4,* N6)

Some participants mentioned the fact that sometimes the patient ends up being rude because of their medical condition. Below are the responses;

*‘‘I remember treating a very rude patient. This patient was very rude to me because he was in pain. In fact, I was very angry but I remained calm and did not react. But after serving his pain medications, he became calm. So, sometimes the condition of the patient causes the patient to be rude’’.* (FG 2, N6)

#### Negative attitude from patients

The respondents of this current study acknowledged the fact that some of the patients exhibited certain behaviors which made it difficult for them to communicate with them. Below are some narrations;

*‘‘There are some patients when you ask them questions, they refuse to respond, so this can hinder your therapeutic interaction. Also, some patients are unwilling to talk and they can reject treatment or refuse to take the medicine. When you are talking to them, they won’t mind you as such you can talk to the person the whole shift but the person will not be willing to talk or hmmm’’. (*FG1, N1)

*‘‘Others too answer in a rude manner so even if there is something you need to tell them, due to their rudeness, you are unable to establish that therapeutic interaction with the patient, you may want to go and come back when the patient is calm’’.* (FG2, N2)

Some nurses shared their personal encounters with some patients who were rude to them and how they managed the situation.

*‘‘Okay, sometimes when the patient comes, if he/she is in pain or very anxious, he or she ends up giving you some bad attitude. A patient was admitted to my ward recently. When she came, she looked very anxious. I went closer to her to try and help allay her anxiety. This patient for no reason started raining insults on me. I was worried but I had to take it cool and find out what is wrong, not knowing she had issues with the OPD nurses and she is transferring her anger onto me’’.* (FG4, N6)

#### Increased workload/stress

Some participants recounted how increased workload and burnout hinder their therapeutic communication practice with their patients. Below are some responses;

*‘‘workload. Workload sometimes makes health personnel become snobbish and ignore patients’ concerns because they feel they have a lot to do and they cannot take the time to listen to everyone in detail about their concerns. So, the workload makes nurses rush even when listening to patients’ concerns’’.* (FG2, N5)

*‘‘I will say the workload of the nurse will determine how the nurse will communicate when the place is very busy and the nurses are trying to concentrate or there is an emergency. The nurses might spend all the time attending to emergency situations and will have no time to communicate with other patients’’.* (FG5, N1)

#### Family interference

There are times when family interference disrupts the nursing care of the patient. Participants in the study revealed instances of therapeutic communication was not effective due to family interference. Below are some responses;

*‘‘Sometimes, when you’re taking care of a patient, some relatives of the patient interfere and end up telling you what to do and what not to do. This is very a major challenge and it makes it difficult for us to have effective therapeutic communication with our patients’’. (FG1,* N3)

*‘‘Most times, family members would want to interfere with everything thus making it difficult to communicate with the patient. Sometimes, the patient is willing to do what he or she has been asked to do; But the relatives would end up changing the mindset of the patient. This attitude of the relatives greatly affects therapeutic communication and effective nursing care’’. (*FG5, N6)

A participant narrated an encounter she had with a relative of a patient who interfered with the care provided and how she salvaged the situation.

*‘‘Hmmmm, the family hmmm. One time, I was taking care of a patient, the patient was to take some medications. I went to the patient to administer the medication. Out of nowhere, a relative of the patient came and said that he won’t allow his brother to take the medication. I explained the importance of the medication to him but he still didn’t understand. He ended up creating a scene. My sister, it was very bad. But in the long run, the patient ended up taking the medication. So, the family sometimes interrupts our therapeutic communication with their patient’’. (*FG 4, N4)

#### Discriminatory attitude of nurses

Discrimination during nursing care could be exhibited by both the nurse and the patients. Participants in the study revealed that some patients showed discriminatory attitudes towards the nurses during nursing care as they preferred some particular nurses to provide care for them. Below are some responses;

*‘‘Hmmm, some patients behave in an unusual manner. They want some particular nurses to take care of them. When those nurses are not around, they won’t even allow you to get closer to them. When you want to even give them their medication, they’ll tell you that, they want that particular nurse to be the one to give them their medication. No matter the number of times you try to convince them, they’ll not listen until they see that particular nurse’’.(* FG3,N3)

*‘‘I remember treating one particular patient. It was time to dress his wound. I sent my pack and everything to his bedside to dress his wound. I got there and informed him of my intention. He asked me about the whereabouts of my other colleague. I told him that she is not on duty today and that she’ll report tomorrow. This patient said he’ll wait for her to come. I tried explaining to him that his wound needs to be dressed but he declined. This patient said he only wants that nurse to dress up his wound.’’.* (FG5,N6)

Other participants also reported that sometimes some nurses also exhibit these discriminatory attitudes.

*‘‘Some nurses discriminate among the patients. Some of the nurses do not want to associate themselves with patients with some kind of condition. For instance, some nurses always shy away from patients with diabetic wounds. They complain of the stench that comes out from it and they don’t want to associate themselves with them. That is very bad’’ (FG 2,* N6)

*‘‘Hmm, the discrimination among patients from nurses is very bad. Most nurses wouldn’t want to associate themselves with patients with poor backgrounds. They always want to associate themselves with those from rich backgrounds because they sometimes get tips from them. This attitude is very bad’’. (FG4.* N1)

## Discussion of findings

### Therapeutic communication practices

The recent study revealed that manners accompanying nurse-patient interaction could have an impact on communication. Some manners identified to positively impact communication included, approaching the patient with a smile while screaming and chewing gum were found to interfere with therapeutic communication practice. The results are consistent with previous research findings that nurses who communicate politely and respectfully with their patients receive cool responses and behaviors from their patients, and vice versa for nurses who behave poorly [36, 37]. Patients not only consider words spoken during caregiver-patient interactions but also observe some nonverbal behaviors that accompany the caregiver's spoken words. Therefore, it is important for caregivers to ensure that spoken language matches the non-verbal behavior of others to prevent misunderstandings. This is because these negative behaviors can distort the therapeutic relationship between caregivers and patients and adversely affect patient health outcomes. When nurses exhibit negative attitudes toward patients, the patients are unable to communicate freely**,** hence approaching patients in the right manner fosters therapeutic communication.

The use of therapeutic touch was one of the key tools participants in this study used during communication. Many participants verbalized that gentle back rubs and taps on the shoulders helped reduce anxiety and pain felt by patients. This finding was consistent with studies showing that therapeutic physical contact had a positive impact on the aggressive management of pain-related parameters in cancer patients [38]. Touch is an effective tool that helps to communicate love, care, and attention. It is also used during examinations and as a form of pain reliever. Caregivers are therefore encouraged to use it appropriately while caring for the patient as it is an active part of the therapeutic relationship. Consent must first be obtained from the patient before practicing therapeutic touch to prevent being charged with battery.

The current study further revealed the impact of positive reassurance on nurse-patient relationships and the need to avoid giving false hopes. This finding is consistent with findings from another study that showed that nurses used language, gestures, education, and relationship-building to reassure patients [39]. Another study also revealed the need for nurses to remain calm when their patients are angry [40]. Reassurance should be used tactfully since, offering false reassurance goes against the principle of veracity which could lead to mistrust, conflicts, and non-adherence to treatment. Hence, nurses should choose their words carefully when reassuring patients, and provide them with facts about their condition and what is being done to manage it. This will give them a sense of hope, maintain trust, and increase patients’ cooperation and adherence to treatment. Nurses should also maintain a calm demeanor when attending to patients in critical conditions since it helps communicate a sense of hope and put patients and their relatives at ease.

The involvement of patient relatives in a nurse-patient therapeutic relationship was mentioned by the study participants as one of the effective tools in therapeutic communication. They mentioned that working with the patient’s family is key, as family members are responsible for providing financial and other support to the patient. Results from previous studies have shown that nurses engage patients’ families in therapeutic communication and care, which reduces family concerns and fears and facilitates patient recovery [41, 42]. Families most of the time are involved in rendering financial, emotional, social, and spiritual support when a member is admitted, hence their concerns and suggestions should be taken into consideration. Recognizing their contributions motivates them to give their maximum support by ensuring that, their relatives adhere to all instructions and advocate for them where necessary.

The majority of the participants from this present study revealed that patients’ condition (the medical diagnosis, and pain complaints) was one of the major factors that hinders the effectiveness of therapeutic communication. This finding agrees with the results of a survey that found that patients’ anxiety, pain, and physical discomfort were the most important patient-related factors that pose barriers to effective nurse-patient communication [23, 42]. This means that nurses should try to assess the patient's condition in order to identify and address the patient's problem. This is because it can interfere with therapeutic communication. At the beginning of the interaction, the nurse is expected to ask about the patient's concerns. This helps patients be prepared to communicate, ensures openness, and fosters collaboration. This smoothens caregiver-patient relationships and improves patient outcomes. It also helps patients communicate challenges early for prompt action to be taken to prevent complications.

Negative attitude exhibited by some patients (such as refusing treatment, insults, and being uncooperative) was also identified to hamper therapeutic communication in this current study. A similar finding from a study revealed that nurses exhibit poor attitudes toward some patients and hence fail to communicate treatment regimen guidelines to them efficiently [23, 43]. It is important to note that most of the patients exhibit such attitudes when they are not satisfied with the care provided or when they are not seeing improvement in their conditions. Therefore, nurses and other health care providers should appreciate positive relationships between nurses and patients to reduce such cases. Nurses are expected to assess patient satisfaction with care, apologize for mistakes, and reassure patients that they will make amends. This can reduce misunderstandings and conflicts between patients and caregivers.

High workload and limited time were outlined by the majority of the participants in the current study to contribute to poor therapeutic communication between the nurse and the patient. This finding is in tandem with a study carried out at the Kumasi South Hospital to ascertain the perceived barriers to effective therapeutic communication among nurses and patients where most of the respondents (84.7%) denoted that being overworked was the main barrier to effective communication [23]. This is a major challenge that could be overcome by addressing staffing issues, nurse physical and emotional burnout, and incentivization. The government, stakeholders, and other non-governmental organizations are expected to find innovative ways to solve issues of understaffing in nursing and reduce stress among nurses since they form the majority of the healthcare workforce and spend much time with their patients. Hence, their satisfaction could go a long way to ensure that quality care is delivered and nurse-patient interaction is effective.

Some participants in this study indicated that family members of patients sometimes interfered with therapeutic communication by asking them to decline the treatment offered to them. Similarly, one study found that family members showed up at odd times, disrupting nursing and medical care and causing discomfort to other patients [43]. This in turn could result in a misunderstanding that affects therapeutic communication with patients and relatives. Nevertheless, nurses are to handle any misunderstanding that stems from patient relatives amicably as family involvement is a critical component of patient-centered care that impacts the quality of care and patient outcome.

In this study, nurses found that some patients preferred certain nurses and therefore refused care from unpreferred nurses. Discrimination and perceived attitudes. Some participants also noted that some patients may refuse treatment, which may ultimately adversely affect patient outcomes. A somewhat similar finding was seen in a different study where nurses noted that they faced racial discrimination. Most of the nurses who were African Americans stipulated that some patients would prefer not to communicate with them nor will they receive any form of treatment as a result of their race [44].

## Conclusion

Several communication practices impede therapeutic nurse-patient communication. This included nurses’ manner of communication, the use of therapeutic touch, positive reassurance, and nurses’ demeanor. Polite and respectful nurse-patient communication is noted to elicit calm responses and positive behavior from patients, hence the need to reinforce positive communication practices to improve therapeutic nurse-patient interaction and patient satisfaction.

### Study limitation

This study focused on the perspective of nurses only hence future researchers could analyze nurses’ and patients’ perspectives together.

## Supplementary Information

**Additional file 1.**

## Data Availability

All supporting data have been made available and have been uploaded with the manuscript.

## Abbreviations

DHRCIIRB: Dodowa Health Research Center Institutional Review Board
FGDs: Focus Group Discussions
KATH: Komfo Anokye Teaching Hospital
U.K: United Kingdom
